# Development of stock correlation networks using mutual information and financial big data

**DOI:** 10.1371/journal.pone.0195941

**Published:** 2018-04-18

**Authors:** Xue Guo, Hu Zhang, Tianhai Tian

**Affiliations:** 1 School of Statistics and Mathematics, Zhongnan University of Economics and Law, Wuhan, China; 2 School of Mathematical Sciences, Monash University, Clayton, VIC, Australia; Utah State University, UNITED STATES

## Abstract

Stock correlation networks use stock price data to explore the relationship between different stocks listed in the stock market. Currently this relationship is dominantly measured by the Pearson correlation coefficient. However, financial data suggest that nonlinear relationships may exist in the stock prices of different shares. To address this issue, this work uses mutual information to characterize the nonlinear relationship between stocks. Using 280 stocks traded at the Shanghai Stocks Exchange in China during the period of 2014-2016, we first compare the effectiveness of the correlation coefficient and mutual information for measuring stock relationships. Based on these two measures, we then develop two stock networks using the Minimum Spanning Tree method and study the topological properties of these networks, including degree, path length and the power-law distribution. The relationship network based on mutual information has a better distribution of the degree and larger value of the power-law distribution than those using the correlation coefficient. Numerical results show that mutual information is a more effective approach than the correlation coefficient to measure the stock relationship in a stock market that may undergo large fluctuations of stock prices.

## Introduction

Complex network analysis in recent years has become a powerful tool to investigate challenging problems in a wide range of research areas. A complex network is defined as a system with a large number of nodes and relationships between these nodes [1]. A variety of methods have been applied to study complex networks in biology, social sciences, finance and engineering. Among them, the stock network is an important financial system [2]. Each node in a stock network stands for a stock, and the edge connecting a pair of stocks represents the correlation between the prices of these two stocks. The stock networks have been used to observe and analyze the dynamics of the stock market as well as make predictions of future prices [3].

To build stock networks, the commonly used algorithms include the Minimum Spanning Tree (MST) [4], the Planar Maximally Filtered Graph (PMFG) [5, 6], and the Correlation Coefficient Threshold Method [7]. In addition, the Dynamic Spanning Tree (DST) has been proposed to study stock networks, but it was found that the DST shrank over time [8]. Among them, the MST network may have the simplest structure. For example, a network was developed for the New York stock market by using MST [7]. In addition, Gilmore et al. analyzed the stabilization of commodity clusters based on MST [9]. However, the real market network showed a more structured hierarchy of the stock importance that was not captured by this developed model. On the basis of the DAX30 stock price data, a comparison study employed these three methods to analyze the underlying economic reasoning for a number of aspects regarding the network structure [10]. Numerical results suggested that any single method might not be able to extract all the economic information from the correlation coefficient matrix [11]. Thus the topological analysis for the correlation network is a powerful method to search for the economic factors affecting stock prices [12].

An important question in the stock network analysis is how the price of one stock is influenced by the economic factors and prices of other stocks. This influence is closely related to the financial policies, national economic growths and the performance of industrial sectors. In addition, the stock price of a company is based on its performance and prospects of future development. A number of research works have been conducted in recent years to analyze the behavior of the Chinese stock network, which is also closely related to the monetary and austerity policies. As an important growing market, the Chinese market exhibits much stronger correlations than the developed markets [13]. Further analyses based on the high-frequency stock returns found that the Chinese stocks had different average correlation strengths during different financial time periods [14]. In addition, the dynamic analysis suggested that the systemic risk varied in the periods of two market crashes in 2001 and 2008 and other calm time periods [14, 15]. The spatial structure of stock interactions in the Shanghai and Shenzhen stock markets in China suggested that the prominent sector structures existed in a number of subsectors [16]. Further research works have also been conducted to explore the network robustness with regard to the random fault and network fragility by the intentional attacks [16, 17].

Since the globalization of the world economy, the financial markets in the world have been connected to each other. The networks of international stock markets were developed using 83 stock market indices in a diversity of countries and correlation-based measures [18]. A comprehensive study for the stock exchanges located all over the world showed that the correlation among market indices presented both fast and slow dynamics [19]. Research results also suggested that stock networks satisfied the small-world and the scale-free properties [20, 21]. The market performance during financial crises is an important topic in this research area [14]. It has been shown that stock networks took on more concentrated topological structure in financial crises than other time periods [22]. About 500 stocks from the S&P have been used to study the market structure and dynamic trend during the financial crises [23]. Based on the New York Stock Exchange data, it has been verified that the influence posed on all stocks occurs almost simultaneously whether it is from economy or from politic [16]. In addition to the correlation analysis, new concepts and methods such as the partial correlation network and the lead-lag relationship, have been introduced to measure the relationship between stocks [24, 25].

Among these studies, the Pearson correlation coefficient is the dominant tool to measure the relationship between two stocks [26]. However, this approach can only measure linear relationships. Mutual information is a measure of statistical independence between two random variables, and it is a more general approach for measuring nonlinear relationships. It can be used to identify the relationship between data sets that are not detected by the commonly used linear measure of correlation [27]. Thus it has a wide range of applications, including the independent component analysis [28] and the analysis for both small and high-dimensional data sets [29–31]. Mutual information comes from Shannon’s entropy theory, and it is unique in its close ties to Shannon’s entropy. However, it is also true that the estimation of mutual information is not always easy. Thus the estimation of mutual information is an important work in information theory [32, 33]. Pluim et al. gave an algorithm to compute mutual information for high-dimensional variables and applied it to medical image registering [34]. It has been shown that the network based on mutual information could replace the network using the correlation coefficient [35]. Although mutual information has been used to develop genetic regulatory networks recently [29, 35], the stock network based on mutual information is still at the early developmental stage. Only the partial mutual information and mutual information rate have been used to compare with the correlation coefficient for developing stock networks [36, 37].

To address the issue of the nonlinear correlation, this work proposes a novel framework to develop stock networks by using mutual information. The stock price data from the Shanghai Stocks Exchange (SSE) are used to demonstrate the effectiveness of this new approach. The remaining part of this paper is organized as follows. Section 2 discusses the computation of mutual information and MST for developing stock networks. In Section 3, we develop two stock networks using mutual information and the Pearson correlation coefficient, respectively, and finally study the topological properties of these networks.

## Methods

### Mutual information

Mutual information from entropy theory is a generalized correlation measurement. According to Shannon’s entropy theory [32], the entropy of a discrete random variable *X* is defined by

$$\begin{array}{ccc} H(X) & = & -\sum_{i}p\left(x_{i}\right)log_{2}p\left(x_{i}\right), \end{array}$$ (1)

where *p*(*x*_*i*_) is the probability distribution of *X*. Entropy is used to measure the uncertainty of a random variable, which is equivalent to the quantity of information it owns. For two-dimensional random variables (*X*, *Y*), the joint entropy is given by

$$\begin{array}{ccc} H(X,Y) & = & -\sum_{i}\sum_{j}p\left(x_{i},y_{j}\right)log_{2}p\left(x_{i},y_{j}\right), \end{array}$$ (2)

where *p*(*x*_*i*_, *y*_*j*_) is the joint probability distribution of (*X*,*Y*). The mutual information of *X* and *Y* is then defined by

$$\begin{array}{ccc} I(X,Y) & = & H(X)+H(Y)-H(X,Y), \end{array}$$ (3)

which can be interpreted as the information that *X* and *Y* share. In addition, mutual information can be defined as

$$\begin{array}{ccc} I(X,Y) & = & H(X)-H(X|Y), \end{array}$$ (4)

where *H*(*X*|*Y*) is the conditional entropy of *X* under the condition *Y*, which is defined as

$$\begin{array}{c} H\left(X|Y\right)=-\sum_{i}\sum_{j}p\left(x_{i},y_{j}\right)log_{2}p\left(x_{i}|y_{j}\right), \end{array}$$ (5)

where *p*(*x*_*i*_|*y*_*j*_) is the conditional probability. In this definition, mutual information is regarded as the uncertainty of random variable *X* removed under the condition *Y*. Mutual information *I*(*X*, *Y*) = 0 holds if and only if *X* and *Y* are independent. We can normalize mutual information into the interval [0, 1] by using

$$\begin{array}{ccc} \text{NMI}(X,Y) & = & \frac{2I(X,Y)}{H(X)+H(Y)}. \end{array}$$ (6)

From the above definitions, we need the probability distributions to exactly compute mutual information. Since it is difficult to obtain such distributions for complex problems, we use a numerical method to compute the mutual information of stock returns [29]. Considering a network of *n* stocks with prices in *d* trading days, denote *P*_*i*,*t*_ and *R*_*i*,*t*_ as the closing price and log-return of stock *i* at day *t*, respectively, given by

$$\begin{array}{ccc} R_{i,t} & = & ln\frac{P_{i,t}}{P_{i,t-1}},\left(t=2,...d;i=1,2...,n\right). \end{array}$$ (7)

Hence, for stock *i*, we set the corresponding log-return interval as [*minR*_*i*,*t*_, *maxR*_*i*,*t*_] and uniformly divide it into *k* sub-intervals. We compute the frequency of stock *i* falling into the sub-interval *q*, and approximate the probability by using the frequency

$$\begin{array}{c} p_{i,q}\approx \frac{f_{i,q}}{d},\left(i=1,2...,n;q=1,2...,k\right). \end{array}$$ (8)

The entropy of stock *i* then is approximated by

$$\begin{array}{c} H\left(S_{i}\right)=-\sum_{q=1}^{k}p_{i,q}log_{2}p_{i,q}. \end{array}$$ (9)

To compute the joint entropy of stocks *i* and *j*, we uniformly divide the square of log-return [*minR*_*i*,*t*_, *maxR*_*i*,*t*_] × [*minR*_*j*,*t*_, *maxR*_*j*,*t*_] into *k* × *k* bins. Denote $\frac{f_{i,j,q,r}}{d}$ as the frequency of joint log-returns falling into the bin (*q*, *r*), which can substitute the joint probability distribution with

$$\begin{array}{c} p_{i,j,q,r}\approx \frac{f_{i,j,q,r}}{d},\left(i,j=1,...,n;q,r=1,...,k\right). \end{array}$$ (10)

The joint entropy of stock *i* and *j* can be approximately computed by

$$\begin{array}{c} H\left(S_{i},S_{j}\right)=-\sum_{q=1}^{k}\sum_{r=1}^{k}p_{i,j,q,r}log_{2}p_{i,j,q,r}, \end{array}$$ (11)

and the mutual information of stock *i* and *j* is estimated by

$$\begin{array}{ccc} I(S_{i},S_{j}) & = & H\left(S_{i}\right)+H\left(S_{j}\right)-H\left(S_{i},S_{j}\right). \end{array}$$ (12)

When computing the normalized mutual information by using (11 and 12), we can choose a different number of bins. To test the influence of bin number on the value of mutual information, we calculate the value using 10×10, 15×15, 20×20 bins. For the same stock pair, we find that the largest difference of the values between 10×10 and 15×15 bins, and that between 10×10 and 20×20 bins are 0.0073 and 0.0107, respectively. This result shows that once the bin number is adequately large, any further increase of the bin number has not much influence on the accuracy of mutual information. Thus, we use 10×10 bins in this study.

On the other hand, the correlation coefficient of stocks *i* and *j* is computed by

$$\begin{array}{ccc} \rho_{i,j} & = & \frac{\sum_{t=1}^{d}\left(R_{it}-\bar{R_{i}}\right)\left(R_{jt}-\bar{R_{j}}\right)}{\sqrt{\sum_{t=1}^{d}{\left(R_{it}-\bar{R_{i}}\right)}^{2}\sum_{t=1}^{d}{\left(R_{jt}-\bar{R_{j}}\right)}^{2}}}, \end{array}$$ (13)

where $\bar{R_{i}}$ is the average log-return of stock *i* over *d* trading days.

In a network, the distance between nodes must be given by a metric. In the network based on the Pearson’s correlation coefficient, a usual metric is

$$\begin{array}{c} d_{\rho }\left(X,Y\right)=\sqrt{2(1-\rho_{X,Y})}, \end{array}$$ (14)

which transfers the correlation coefficient range [−1, 1] into the interval [0, 2]. The distance between nodes based on mutual information is defined by

$$\begin{array}{c} d_{M}\left(X,Y\right)=H\left(X\right)+H\left(Y\right)-2I\left(X,Y\right). \end{array}$$ (15)

We can verify that it satisfies the non-negative, symmetric and triangle inequality properties. In addition, this metric has a normalized version

$$\begin{array}{ccc} D(X,Y) & = & 1-\frac{I(X,Y)}{H(X,Y)}. \end{array}$$ (16)

Similarly, the distance of stocks *i* and *j* in the stock network is

$$\begin{array}{ccc} D(S_{i},S_{j}) & = & 1-\frac{I(S_{i},S_{j})}{H(S_{i},S_{j})}. \end{array}$$ (17)

### Minimum Spanning Tree

We will use the MST method to build the stock network. Here, a graph is denoted by *G*(*V*, *E*), where *V* = {*v*_1_, …, *v*_*n*_} is the set of nodes, *E* = {*e*_1_, *e*_2_, … *e*_*m*_} is the set of edges and the edge (*v*_*i*_, *v*_*j*_) connects nodes *v*_*i*_ and *v*_*j*_. If the edge (*v*_*i*_, *v*_*j*_) is undirected, the graph is called undirected graph. A path is a graph which has finite distinct nodes and each edge connects two adjacent nodes. If the nodes belonging to a path are different, the path is called a simple path. If two endpoints are equal, the path is called a loop. When each edge has a weight, the graph is called a weighted graph. For an un-weighted graph, the length of a path is the number of edges. For a weighted graph, the length of a path is the sum of weights. In an undirected graph, if there is a path linking endpoints *v*_*i*_ and *v*_*j*_, these endpoints are called connective. If any two nodes are connective, the graph is connective.

A tree is a connected acyclic graph. A MST is a spanning graph with a minimal sum of weights. For the stock network, we use the distance between two stocks as the weight of an edge. There are two popular algorithms for constructing an MST. Among them, the Kruskal algorithm ranks the weights of edges in an ascending order and adds the next edge with the smallest weight if this addition does not create a cycle. The complexity of the Kruskal algorithm is *O*(*mlnm*) where *m* is the number of edges. On the other hand, the Prim algorithm grows the spanning tree from a given node, and iteratively adds the shortest edge from a node in the network to the node that has not been reached yet, until all the nodes are reached. The complexity of the Prim algorithm is *O*(*n*^2^) where *n* is the number of nodes. Generally, the Kruskal algorithm is suitable for sparse networks, while the Prim algorithm is better for dense networks.

In this work, we use the Prim algorithm to construct stock networks. Suppose that *G*(*V*, *E*) is a weighted undirected connective graph with *n* nodes. The MST, denoted as *T*(*TV*, *TE*), is constructed by:

1. *TE* is empty, *TV* = *u*_1_, *u*_1_ ∈ *V*
2. For all edges with *u* ∈ *TV*, *v* ∈ {*V* − *TV*}, find the shortest edge (*u*, *v*). If the network is not cyclic, add *v* into *TV* and add (*u*, *v*) to *TE*. Otherwise, reject this edge and then consider the next shortest edge.
3. Repeat step 2 till *TV* = *V*.

## Results

### Chinese stock market

There are more than 2000 companies traded at the Shanghai Stock Exchange (SSE). In this work, we consider a subsystem that is related to the real estate industry. Currently the real estate industry is a very important part of the market economy in China. A number of stocks in the financial, banking and chemical sectors have much influence on the stock market. We choose stocks from companies related to the real estate, chemical industry, automobile, banking, building materials, cement, non-banking financial, as well as iron and steel sectors. We remove the stocks that have poor business performance and face the risk of delisting. The Chinese stock market is a growing market. Each year a number of companies are added to the market. Thus we cannot use the market data over a long time period. Otherwise, a proportion of stocks will have to be excluded from our study because of the incompleteness of data. We finally select 280 stocks with 734 trading days between 04/01/2014 and 30/12/2016. During this time period the stock index experienced substantial fluctuations from stagnation (01/2014-06/2014), sharp increase (07/2014-06/2015), crash (06/2015-07/2015) and recovery (07/2015-12/2016), which are called the four time periods in the following study. This is a good test system to examine the effect of different measures for the stock relationship. The stock index during this time period is given in S1 Fig.

### Comparison of robustness of two measures

**Fig 1:**
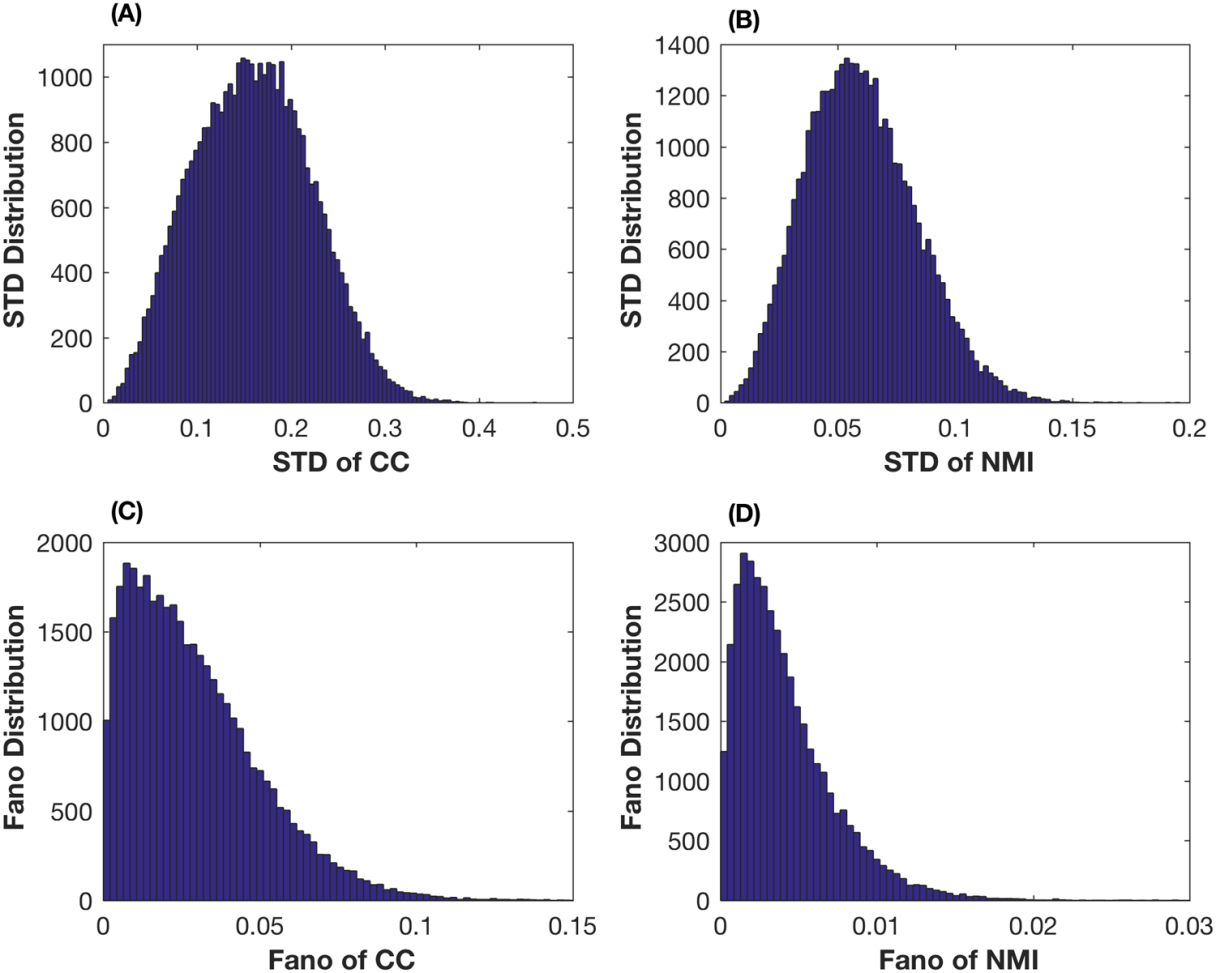
Comparison of STD and Fano factor values for the prices of stock pairs using the correlation coefficient and mutual information.

We first test the robustness property of the correlation coefficient and mutual information for measuring stock prices with large variations. For each measure, we first calculate five values based on the stock prices in the four time periods as well as the prices in the whole time period. Then we calculate the standard derivation (STD) of these five values. Fig 1 gives the STD values of 39060 (namely $C_{280}^{2}$) stock pairs. The STD values of the correlation coefficient in Fig 1A range from 0.0048 to 0.4472, while those of mutual information in Fig 1B are between 0.015 and 0.1817. To remove the influence of the mean, we further calculate the Fano factor, given by

$$\begin{array}{ccc} F & = & \frac{\sigma^{2}}{\mu }, \end{array}$$ (18)

where *μ* and *σ*^2^ are the mean and variance of the five values for each stock pair. Fig 1D shows that the range of the Fano factor values for mutual information is much smaller than that of the correlation coefficient in Fig 1C. These results suggest that mutual information is a more robust measure than the correlation coefficient for the stock price data with large variations.

**Fig 2:**
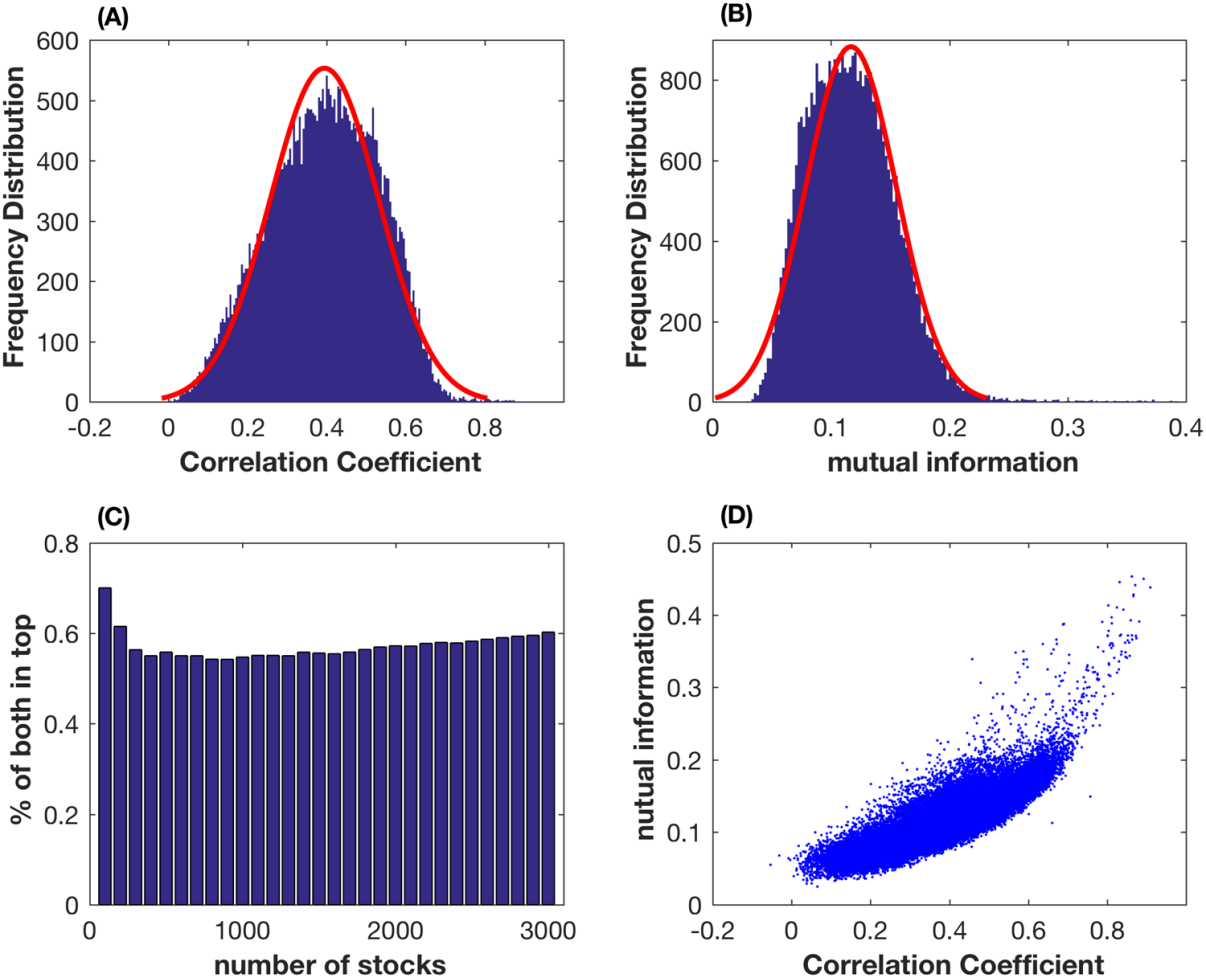
Comparison study of the correlation coefficient and normalized mutual information. (A) Frequency distribution of the correlation coefficient values. (B) Frequency distribution of normalized mutual information values. (C) Percentage of stock pairs that are the top pairs when different numbers of top stock pairs in both the correlation coefficient and mutual information measures when different numbers of top stock pairs are considered. (D) Normalized mutual information values against the corresponding values of the correlation coefficient.

Based on the stock price data in 2014∼2016, Fig 2A shows that all the correlation coefficient values fall into the interval [-0.0532, 0.9096] and the majority of values are between 0.2 and 0.6. In addition, 166 pairs of stocks have high correlation coefficients that are above 0.7, but only 4 pairs have negative correlation coefficients with small absolute values. For the normalized mutual information, Fig 2B suggests that the values fall into the interval [0.0414, 0.5815]. For the studied stocks, 82 pairs have normalized mutual information that is above 0.4, 1232 pairs fall into the interval [0.3,0.4], and there are 19497 pairs whose normalized mutual information is between 0.2 and 0.3.

### Comparison of top stock pairs

To compare the effectiveness of mutual information and the correlation coefficient, we first resort the values of these two measures in descending order separately. In the top 100 pairs of each measure, there are 70 pairs of stocks that appear in both measures. If we consider the top 200 pairs, 124 pairs of stocks have both large values of mutual information and the correlation coefficient. When comparing more top stock pairs, Fig 2C shows that about 60% of the top stock pairs appear in both measures. Although Fig 2D suggests that a linear relationship may exist between the values of normalized mutual information and those of the correlation coefficient, there are still substantial variations between the ranks of stock pairs that are derived from these two measures.

**Fig 3:**
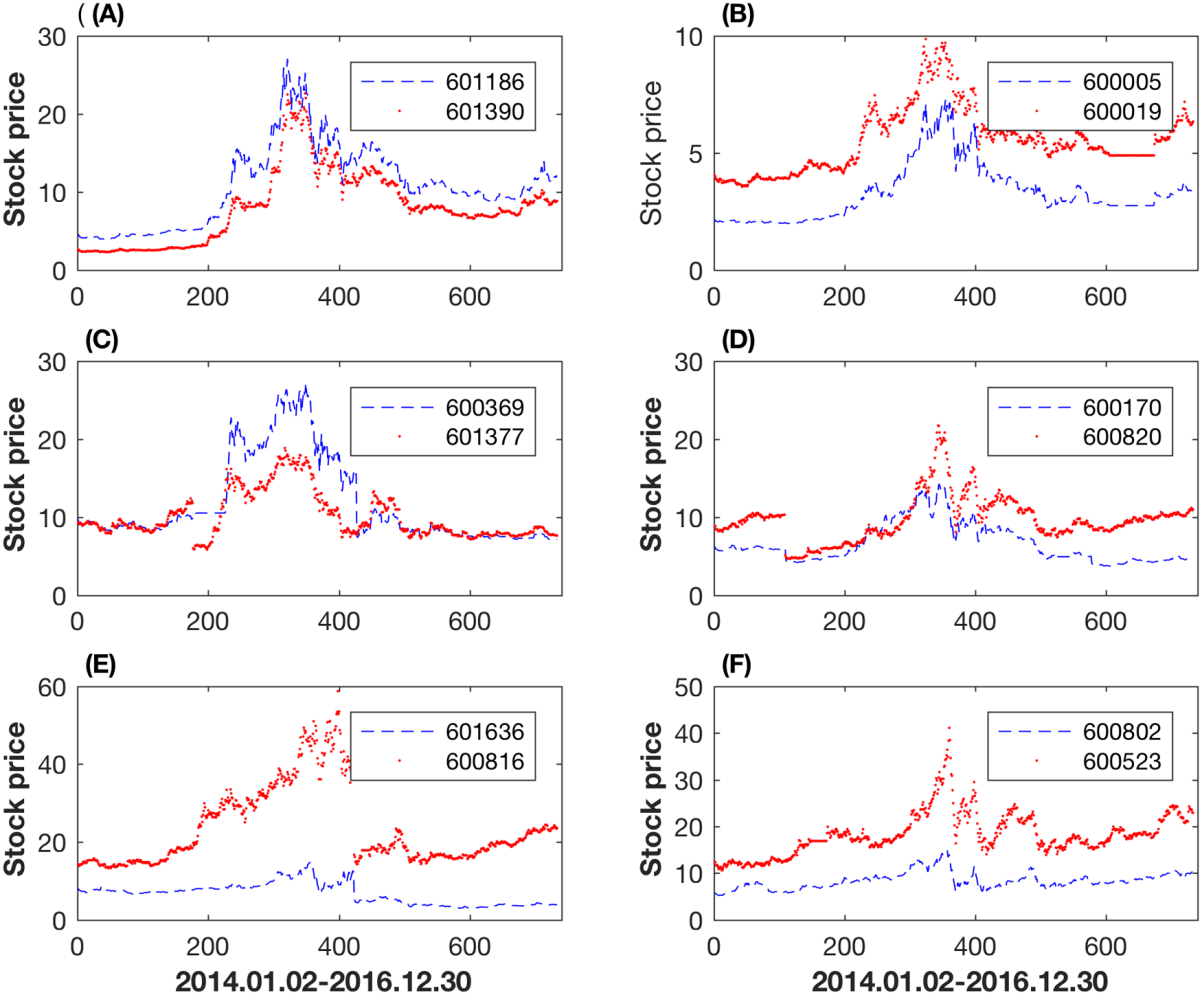
Daily closing prices of six stocks pairs. (A,B) Stock pairs having large values of both the correlation coefficient and mutual information; (C,D) Stock pairs having large value of mutual information but relatively small value of correlation coefficients; (E,F) Stock pairs having large values of correlation coefficients but relatively small values of mutual information.

Based on the values of two measures, all the stock pairs can be classified into four types. The first type has large values of both mutual information and the correlation coefficient. For example, the pair of China Railway Construction Corporation Limited (601186) and China Railway Engineering Corporation (601390) has the largest values in both measures. These two companies are in the same sector with similar primary business activities that lead to almost the same price dynamics. The stock prices of these two companies in Fig 3A are very similar to each other. Fig 3B shows the prices of Wuhan Iron and Steel Company (600005) and Baoshan Iron and Steel Company (600019). This stock pair ranks the sixth in the mutual information measure and seventh in the correlation coefficient measure.

In the second type, the stock pairs have large values of mutual information but small values of the correlation coefficient. These stocks can be further divided into two major groups. In the first group, stock prices change with large volatilities. For example, Southwest Securities (600369) and Industrial Securities (601377) in Fig 3C have the similar fluctuation trends, but are not linearly dependent. The stock price of Southwest Securities has nearly vertically declined from the highest price of 25 Chinese Yuan. Its price trend is consistent with the Shanghai Composite Index. This highly nonlinear correlation measured by mutual information cannot be expressed well by the correlation coefficient. In the second group, companies had rationed their shares before the large price movement. One example is the Shanghai Construction Group (600170) and Shanghai Tunnel Engineering Company (600820) in Fig 3D.

In the third type, the stock pairs have large values of the correlation coefficient but small values of mutual information, including the Kibing Group (601636) and Anxin Trust (600816) in Fig 3E, Fujian Cement (600802) and Guihang Automotive Components (600523) in Fig 3F. For these stock pairs, normally one of them has large volatility in price, but the other is relatively stable. Anxin Trust in Fig 3E paid stock dividend on 23/09/2015, and its stock price fluctuated violently before this date. However, the stock price of Kibing Group (601636) was always stable. In the second example, the price of Fujian Cement (600802) was stable at a low level due to its industrial development; however, the price of Guihang Automotive Components (600523) has experienced relatively large fluctuations in Fig 3F. Finally, the fourth type includes stock pairs whose mutual information and the correlation coefficient all have small values. This type of stock pairs is not discussed in this work.

During the developmental process of the Chinese stock market, especially in the studied period of 04/01/2014-30/12/2016, the Chinese stock market underwent violent fluctuations from time to time. Thus it is inappropriate to consider the third type of relationship discussed above, though these stock pairs have large values of the correlation coefficient. However, the second type of relationship is important for the nonlinear correlation between stock pairs. Therefore, in this work we propose to use mutual information to measure the relationship between stocks. For comparison study, we also develop corresponding networks using the correlation coefficient.

### Hierarchical networks

**Fig 4:**
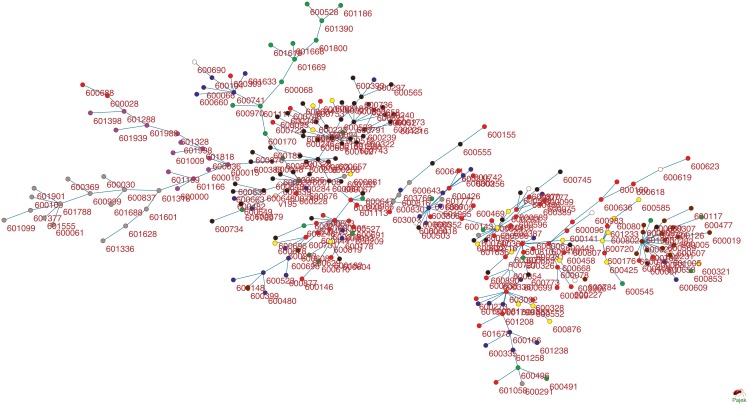
Stocks network based on normalized mutual information using stock price data from 04/01/2014 to 30/12/2016.

Based on the values of mutual information and the correlation coefficient for each stock pair, we next use the MST method to build the undirected weighted network. We label each stock using its corresponding stock code and distinguish stocks in different sectors by using different colors, namely chemical (red), building materials (yellow), ornament (green), automobile (blue), household electrical appliance (white), real estate (black), banking (purple), non-banking financial (gray), and iron and steel (brown). For the network based on mutual information, Fig 4 shows that stocks in the same sector possess certain internal connection properties. Stocks more likely connect stocks within the same sector. Indeed, companies in the same sector provide similar products and service activities, and thus the reaction of their shares to the external influence is also similar to each other.

Fig 4 shows different densities of interconnections between different sectors. According to the interconnection density, the nine sectors in Fig 4 can be classified into three major groups. The first group includes the non-banking financial sector, banking sector, ornament sector, and real estate sector that form the largest group. Sixteen non-banking financial stocks form a sub-group and connect to the network through the Industrial Bank (601166). Banking stocks connect to the network through the Poly Real Estate(600048). Stocks in the financial sector, such as banks and insurance companies, usually offer high dividend yields and their stock prices are low. After the stock market crash in 2015, to maintain the stability of the stock market, banking stocks are usually the primary investment option. This particularity leads to the strong clustering of the stocks in the first group.

The second group includes companies in the automobile, chemical, and household electrical appliance sectors. The major activities of these companies cover a wide range of business activities, and often there are overlaps between the business activities of different sectors. In addition, the correlation between stocks inside each sector is higher than that between different sectors. Stocks in these sectors form small sub-groups inside each sector and are connected to the sub-group in other sectors. For example, the automobile industry has been developing business in new energy and intelligence industry, and the development of the real estate sector also accelerates the growth of the automobile industry. Thus, stocks in the automobile sector connect to stocks in the chemical and real estate sectors. In addition, companies in the chemical industry have a wide range of business activities. There are a number of stocks in this sector forming a few small sub-groups connected to other sectors. Thus, the companies in these three sectors are closely related to each other.

The third group includes companies in the iron and steel as well as building material sectors. The iron and steel stocks connect to the network through stocks in the ornament sector and the center of this sub-group is Shanghai Iron and Steel (600022). Due to the excess of production capacity, the price of iron and steel continues to decline. Companies in this sector have to merge or reorganize in recent years. Companies in the building material sector have low internal relevance without clustering, mainly affected by companies in the real estate sector. Most of the stocks in the third group are on the boundary of the network, namely as the leaf nodes.

As mentioned earlier, the Chinese stock market has experienced different developmental stages during the last three years. To find the influence of different stages on the network structure, we develop two networks for simplicity using the data in 02/01/2014-15/06/2015 and data in 15/06/2015-30/12/2016. Note that 15/06/2015 was the first day of the stock market crash in 2015. S2 and S3 Figs show that these two networks have distinct structures. Before the market crash, the Chinese stock market was a bull market and the majority of stocks went up in the majority of the trading days. Thus, except the banking and non-banking financial sectors, the network in S2 Fig is dense and stocks connect to each other across different sectors. The market effect is more influential on the prices than the internal effect inside each sector. However, the stock prices went down after the market crash. S3 Fig shows that stocks form four major clusters, namely the financial cluster, real estate cluster and two chemical clusters. During this time period, the internal effect in each cluster was more important. Note that in these two networks the banking and non-banking financial sectors are relatively independent of other sectors and have strong internal connectivity.

**Fig 5:**
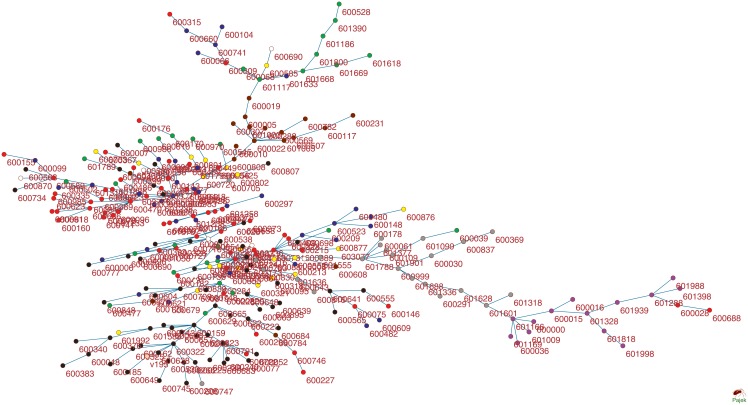
Stocks network based on the correlation coefficient using stock price data from 04/01/2014 to 30/12/2016.

For a comparison study, we also develop a network of these 280 stocks based on the correlation coefficient in Fig 5. Comparing with the stock network based on mutual information in Fig 4, we find that the clusters in Fig 5 are not well organized. For example, stocks in the banking sector do not connect to stocks in the real estate sector at all. In addition, the important nodes in this network do not represent the importance of the corresponding companies in these sectors. We also develop two networks in S4 and S5 Figs using the correlation coefficient based on the data in two different stages, namely stages during 04/01/2014-15/05/2015 and 15/05/2015-30/12/2016.

### Network topological properties

We next investigate the topological properties of the developed networks, including degree, path-length and the power-law distribution. The degree of a node is the number of edges connecting it. A node with a larger degree plays a more important role in the network. According to the distribution of degree in Table 1, we analyze three types of stocks with different degrees. The first type includes important nodes that have degrees of more than 6. When financial news affects the stock market, these stocks react first and the fluctuations of their stock prices influence the stocks near them. All these stocks represent the major companies in their sectors. The second type of stocks have degrees between 2 and 6. These stocks deliver market information along the branches. The third type is the boundary stock with degree 1. The majority of the nodes are boundary nodes in these MST networks. Although the difference between the distributions of these two networks in Figs 4 and 5 is not large, the variance of degrees for the network using mutual information is smaller than that using the correlation coefficient. Finally, connections between nodes in these two networks are highly non-uniform. The network is called scale-free, if very few nodes in a network have a large number of connections but the majority of nodes have small connections. Thus it shows that the two stock networks in Figs 4 and 5 are both scale-free.

**Table 1:** Network topology properties of six networks including networks in Figs 4 and 5 and four networks in S2–S5 Figs.

Network	Degree Distribution			Topologies		
	1	2-6	≥ 7	AD	LD	PW
**MI**	187(66.79%)	82(29.29%)	11(3.93%)	9.14194	23(601058-601099)	2.08
**MIs1**	161(57.50%)	110(39.29%)	9(3.21%)	8.79831	24(600568-600705)	1.82
**MIs2**	182(65.00%)	88(31.43%)	10(3.57%)	9.6154	27(600315-601398)	2.17
**CC**	171(61.07%)	95(33.93%)	13(4.64%)	8.45125	31(600688-600528)	1.98
**CCs1**	175(62.50%)	92(32.86%)	13(4.64%)	8.45125	31(600528-601998)	1.98
**CCs2**	172(61.43%)	94(33.57%)	14(5.00%)	8.65868	26(600568-601939)	1.93

AD: average distance; LD: longest distance; PW: power law exponent; MI, MIs1, MIs2: network using mutual information and the whole dataset(Fig 4), data of stage 1 (S2 Fig) and data of stage 2 (S3 Fig), respectively; CC, CCs1, CCs2: networks using the correlation coefficient and the whole dataset, data(Fig 5) of stage 1 (S4 Fig) and data of stage 2 (S5 Fig), respectively.

**Fig 6:**
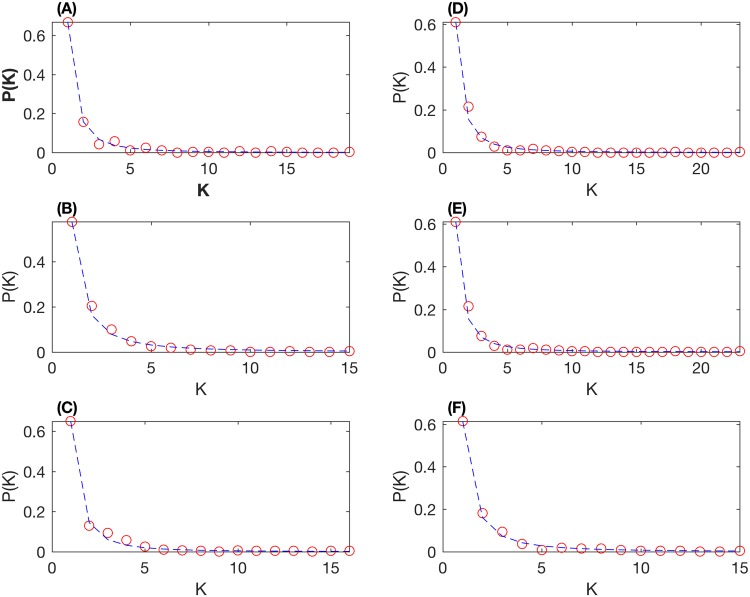
Degree distributions based on normalized mutual information and the correlation coefficient. (A,C,E) Degree distributions based on normalized mutual information with the whole dataset, data of stage 1 and data of stage 2, respectively. (B,D,F) Degree distributions based on the correlation coefficient with the whole dataset, data of stage 1 and data of stage 2, respectively.

To further study the influence of degree, we consider the probability distribution *P*(*k*) of degree *k*. Fig 6 gives the scatter diagrams of calculated frequency. It suggests that, for stock networks in Figs 4 and 5, the probability *P*(*k*) follows the power-law distribution *P*(*k*) ∝ *k*^−*γ*^, where *γ* is the power exponent. In addition, the accumulative influence follows the power-law distribution with *γ* − 1. Based on mutual information, Table 1 shows that the power exponents of the networks based on the whole dataset, stage one dataset and stage two dataset are 2.09, 1.82, and 2.17, respectively. However, when the correlation coefficient is used, the power-law exponents of the networks based on the whole dataset, stage one dataset and stage two dataset are 1.98, 1.98, and 1.93, respectively. From the degree distribution and power-law exponent, the network based on mutual information is more effective to represent the stock system than the correlation coefficient according to these three datasets.

The length of path for a stock pair is the number of intermediate stocks through which these two stocks are connected. The average length of a network can reflect its network size. The average length of the network on the basis of mutual information is 9.1419, which suggests that one stock for affecting another one on average needs to pass through about 10 stocks. The longest path length is 23, which connects the Sailun Group (601015) and Pacific Securities (601058). On the other hand, the average path length of the network using the correlation coefficient is 8.0096.

## Conclusions

In this paper, we have studied the stock relationship network using the data of 280 stocks from the Shanghai Stocks Exchange based on the Pearson correlation coefficient and mutual information. We have compared the stock price patterns for stock pairs that have similar or different value ranks based on mutual information and the correlation coefficient. Compared with the correlation coefficient, our analysis suggests that mutual information is a better approach to characterize the nonlinear dynamic relationship between stock prices when stock market has violent fluctuations. In addition, two stock networks are constructed by using MST. Compared with the network using the correlation coefficient, the network based on mutual information has a better power-law distribution for the degree of stocks; less stocks have large values of degrees; and the majority of stocks have smaller values of degrees. In summary, this work has demonstrated that mutual information is a more effective approach to measure the nonlinear correlation relationship in stock market data. Although substantial progress has been achieved recently to study stock relationship networks, there are still a number of challenging problems in this research area, for example, the analysis of high-frequency trading data and development of dynamic stock networks. These questions will be the potential topics of future work.

## Supporting information

S1 Fig Shanghai Composite Index from 04/01/2014 to 30/12/2016. (JPG)

S2 Fig Stocks network based on normalized mutual information using stock price data from 04/01/2014 to 15/5/2015. (EPS)

S3 Fig Stocks network based on normalized mutual information using stock price data from 15/5/2015 to 30/12/2016. (EPS)

S4 Fig Stocks network based on the correlation coefficient using stock price data from 04/01/2014 to 15/5/2015. (EPS)

S5 Fig Stocks network based on the correlation coefficient using stock price data from 15/5/2015 to 30/12/2016. (EPS)

S1 Table Price data for 280 stocks from the Shanghai Stock Index between 04/01/2014 and 30/12/2016. (XLSX)
