# Supplementary figures and images for: Development of stock correlation networks using mutual information and financial big data
Source: PLoS One. 2018 Apr 18;13(4):e0195941. doi: 10.1371/journal.pone.0195941 (PMC5905993; doi:10.1371/journal.pone.0195941)

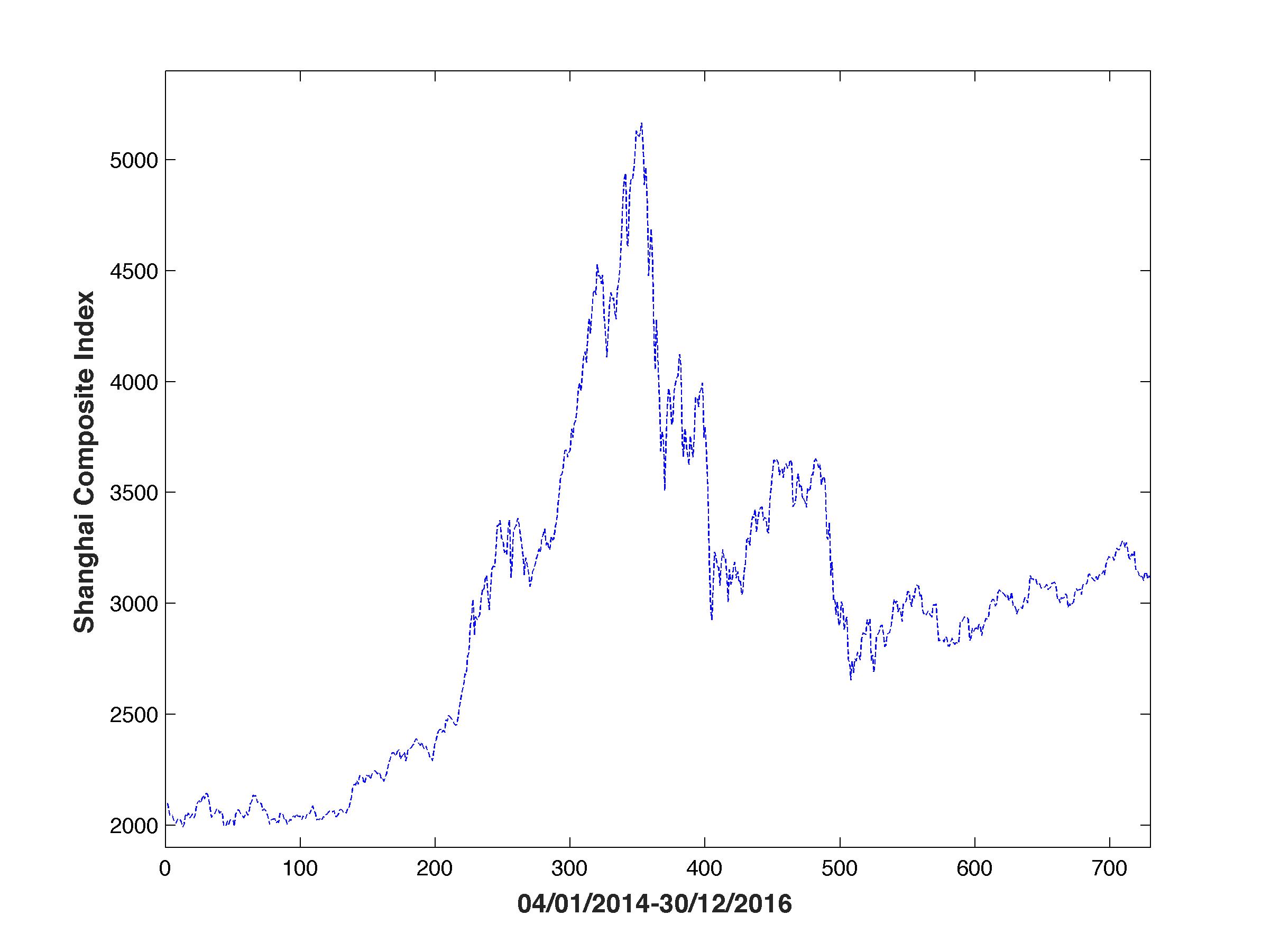

Supplement: S1 Fig — (JPG) [file pone.0195941.s001.jpg]
